# Clinical effectiveness of contemporary dentin bonding agents

**DOI:** 10.4103/0972-0707.73376

**Published:** 2010

**Authors:** Jogikalmat Krithikadatta

**Affiliations:** Department of Conservative Dentistry and Endodontics, Mennakshi Ammal Dental College and Hospitals, Maduravoyal, Chennai 600 095, India

**Keywords:** Bonding agents, clinical trials, noncarious cervical lesions, self-etch adhesives; systematic review, USPHS criteria

## Abstract

**Aim:**

The purpose of this paper is to review the literature on the clinical effectiveness of contemporary resin-based dentin bonding agents primarily focussing on the longevity of restoration.

**Materials and Methods:**

The literature published from June 2004 up to September 2010 was reviewed for clinical trials that tested the effectiveness of dentin bonding agents in the longevity of noncarious class V restoration. Results of each study reported using the USPHS criteria for clinical assessment of restoration were included and tabulated. The American Dental Association guidelines for dentin and enamel adhesives were used as a reference to compare the performance of individual bonding agents. Kruskal–Wallis followed by Mann–Whitney *U* was done to compare the mean Alfa score percentage for the three categories of bonding systems [etch-and-rinse (ER), self-etch primer (SEP), and self-etch-adhesive (SEA)].

**Results:**

A comparison of the mean Alfa score percentages revealed no difference between the ER, SEP, and SEA categories of bonding systems except for marginal adaptation where ER was found to be superior to SEA.

**Conclusion:**

The clinical effectiveness of resin-based bonding agents is comparable among the three categories.

## INTRODUCTION

Contemporary restorative techniques are on the basis of the adhesive properties of tooth-colored resin-based materials. Despite the significant improvements of adhesive systems, the bonded interface remains the weakest area of tooth-colored restorations. The most cited reasons for the failure of adhesive restorations placed with earlier adhesives are the loss of retention and the deficient marginal adaptation.[1,2] Current adhesive systems interact with the enamel/dentin substrate using three different strategies.[3,4]

First is by removing the smear layer (etch-and-rinse (ER) technique) using 30–40% phosphoric acid. Etch-and-rinse system bonding mechanism to dentin is diffusion-based and depends on hybridization of the resin within the exposed collagen mesh as well as into the dentin tubules,[5] creating a micromechanical interlocking of resin within the exposed collagen fibril scaffold. Simplified two-step ER adhesives combine the primer and the adhesive into one application (often referred to as “one-bottle” adhesives).

Second strategy is the “self-etch” adhesives (SEA) which employ the use of nonrinse acidic monomers that simultaneously condition and prime dentin. On dentin, they do not remove the smear layer but hybridize it to the underlying dentin. It impregnates the smear plugs fixing them to the internal tubular walls. There is simultaneous demineralization and infiltration of enamel and dentin to form a continuum in the substrate incorporating the smear plugs in the resin tag.[6] This forms a shallow but a uniform resin-infiltrated interface. Besides simplifying the bonding technique, the elimination of both rinsing and drying steps reduces the possibility of over-wetting or over-drying as they have a negative effect on bonding.[7]

A distinction should be made between “mild” and “strong” SEA. “Strong” SEA have a rather low pH (<1) and have been documented with a bonding mechanism and interfacial ultra-morphology resembling that produced by ER adhesives. Consequently, the underlying bonding mechanism of “strong” SEA is primarily diffusion-based, similar to the ER approach. “Mild” SEA (pH ± 2) only partially dissolves the dentin surface, so that a substantial amount of hydroxyapatite remains available within a submicron hybrid layer. On the basis of the steps of application they can be categorized as: a two-step “self-etch primers” (SEP) and a one-step “self-etch adhesives” (SEA).

Thirdly, glass-ionomers are still considered the only materials that are self-adhering to the tooth tissue.[8] Nevertheless, a short polyalkenoic acid pretreatment is recommended, resulting in a two-step approach. The polyalkenoic acid conditioner cleans the tooth surface; it removes the smear layer and exposes collagen fibrils up to about 0.5–1 mm depth;[9] herein, glass-ionomer components interdiffuse, establishing a micromechanical bond following the principle of hybridization.[3,10] Chemical bonding is additionally obtained by ionic interaction of the carboxyl groups of the polyalkenoic acid with calcium of hydroxyapatite that remains attached to the collagen fibrils.[8]

Although the laboratory testing of contemporary adhesives bonded to sound the tooth substrate under optimal laboratory conditions has been shown to predict clinical effectiveness,[4,11] the ultimate test method to assess bonding effectiveness remains a clinical trial. Peumans *et al*.,[12] in a systematic review, emphasised the need to standardize the conduct and reporting of clinical trials on bonding agents to enable interpretation of the best current evidence. Following are the criteria for conducting, evaluating, and reporting clinical trials undertaken to study the effectiveness of bonding agents: (1) Noncarious cervical lesions (NCCL), (2) Modified United States Public Health Services (USPHS) criteria, and (3) Consolidated Standards of Reporting Trials (CONSORT) statements.

### Non-carious cervical lesions

Noncarious loss of dental hard tissue at the cervical region is used as a clinical model to evaluate the efficacy of dentin bonding agents in nonretentive tooth restorations, as recommended by the ADA.[13] The characteristics of NCCL are:[12] (1) cervical lesions do not provide any macromechanical retention; (2) they require for at least 50% bonding to dentin; (3) when restored, they result in an enamel as well as dentin margin; (4) they are widely available; (5) they are usually found in the anterior teeth or premolars with good access; (6) preparation and restoration of class-V lesions are minimal and relatively easy, reducing somewhat practitioner variability; (7) despite varying cavity configuration factors of class-V lesions, and thus resultant interfacial stress, the mechanical properties of the composite used are relatively unimportant; and (8) ineffective bonding commonly results in restoration loss, which is the most objective evaluation parameter.

### Modified USPHS (Ryge’s criteria)

The modified USPHS/ Ryge criteria[14–17] [Table 1] have been used widely for the clinical evaluation of restorations. Although these criteria do not consider critical issues such as the oral hygiene index and number of decayed, missing and filled teeth, they are the only criteria available for long-term evaluation of restorations. They are considered valid criteria for comparison purposes among studies at different observation periods. The limitation of this system as reported by Hayashi and Wilson[18] is an overlap from Alfa to Bravo ratings for certain characteristics, including marginal adaptation. These variations for some characteristics at various recall examinations need to be interpreted with caution. To facilitate the uniformity among examiners, Fukushima *et al*.[19] emphasized the importance of interexaminer calibration/rating.

**Table 1:** Criteria for modified U.S. Public Health Service and other direct evaluations

Criterion	Test procedure	Score
Retention	Visual inspection with mirror and explore	Alfa: Yes (completely retained)
		Charlie: No (partially or completely lost)
Color match	Visual inspection with mirror at 45 cm	Alfa: No mismatch in the room light in 3 to 4s (margins should be exempted from grading; interfacial staining should not affect grading)
		Bravo: Perceptible mismatch (clinically acceptable)
		Charlie: Esthetically unacceptable (clinically unacceptable)
Marginal discoloration	Visual inspection with mirror at 45 cm	Alfa: No
		Bravo: Superficial staining (removable, usually localized)
		Charlie: Deep staining (not removable, generalized)
Recurrent caries	Visual inspection, mirror, explorer, and radiographs	Alfa: No
		Charlie: Yes
Loss of anatomical form (wear)	Visual inspection with a mirror and explorer, if needed	Alfa: No perceptible wear (or only localized wear)
		Bravo: Generalized wear (clinically acceptable; 50% of margins are detectable; explorer catches going from material to tooth)
		Charlie: Wear beyond dentinoenamel junction (DEJ) (clinically unacceptable)
Marginal adaptation (marginal integrity)	Visual inspection with mirror and explorer, if needed	Alfa: Undetectable
		Bravo: Detectable (V-shaped defect in enamel only; explorer catches going both ways)
		Charlie: Detectable (V-shaped defect to DEJ)
Surface texture	Visual inspection with mirror and explorer	Alfa: Smooth (better than or equal to microfilled composite)
		Bravo: Rougher than microfilled composite
		Charlie: Pitted
Postoperative sensitivity	Questioning the patients	Alfa: None
		Charlie: Some
Other failure		Alfa: No
		Charlie: Yes

## CONSORT

This was initially formulated in 1996[20] and later revised in 2001.[21] The CONSORT statement (or simply CONSORT) comprises a checklist of essential items that should be included in reports of RCTs and a diagram for documenting the flow of participants through a trial. It is aimed at first reports of two-group parallel designs. The objective of CONSORT is to facilitate the critical appraisal and interpretation of RCTs by providing guidance to authors about how to improve the reporting of their trials.

To obtain provisional acceptance, dentin and enamel adhesive materials need to demonstrate that no more than 5% of the restorations are lost and that no more than 5% of the restorations show microleakage at the 6-month recall. To obtain full acceptance, dentin and enamel adhesive materials need to demonstrate that the cumulative incidence of clinical failures after 18 months is less than 10% for lost restorations and 10% for microleakage.[13]

Both laboratory and clinical studies[12,22] have shown that the GIC bonding mechanism is always superior to that achieved by adhesive resins. Hence this group of self adhering materials are not included for comparison in this review. The purpose of this paper was to review the current literature on the clinical effectiveness of resin-based adhesives when used to restore cervical noncarious class-V lesions.

## MATERIALS AND METHODS

Peumans *et al*.[12] published a systematic review on the same research question and the review included clinical trials published until May 2004. Hence the current search included studies published from June 2004 to September 2010.

A PubMed search was conducted with the following key words: dentin bonding agents and noncarious cervical lesions. Specification of the time period in which articles were published, all articles published in English and clinical trials where the limits applied to refine the search. This yielded 366 articles on dentin bonding agents, 37 articles for noncarious cervical lesions, and 20 articles for combined search. Among the 366 articles on dentin bonding agents, 56 articles were short-listed after reading the titles (many included *In vitro* studies). After reviewing the abstracts of the 20 articles in the combined search and 56 articles on dentin bonding agents, 23 studies were included for the systematic review process. Clinical trials of which the data of successive recalls were reported in more than one paper were counted as separate studies.

The Alfa score percentage reported for the bonding agents tested in each study was tabulated under three categories namely, etch-and-rinse (ER), self-etch primer (SEP), and self-etch-adhesive (SEA). Among the parameters of the modified USPHS criteria, marginal discolouration, marginal adaptation, retention, secondary caries, and postoperative sensitivity were considered to be a direct measure of the effectiveness of bonding agents and hence only these parameters were tabulated. Kruskal–Wallis followed by Mann–Whitney *U* was done to compare the mean Afla score percentage for the three categories of bonding agents (ER, SEP, and SEA) using SPSS 15.0 (SPSS Inc, Chicago, USA).

The American Dental Association guidelines for dentin and enamel adhesives were used as a reference to compare the performance of individual bonding agents.

## RESULTS

In general, in the 5.3 years of literature review of PubMed indexed articles, only 23 clinical trials have been reported. A lack of study detail was noted in most of the articles. Long-term follow-up (>5 years) was reported in 8 of the 23 studies. The list of clinical studies with comments on the methodology is shown in Table 2. Six of the 23 studies reported involved the enamel bevel and mechanical preparation of the dentin walls was performed in 16 of the studies reported. Only 5 of the reported studies have mentioned the sample size calculation.

**Table 2:** List of clinical studies published in the past 5 years

Study	Sclerotic dentin	Categorization of lesion size	Sample size calculation	Randomization	Mechanical surface preparation	Enamel bevel	USPHS criteria	Recall rate	Inter-evaluator rating
Van Meerbeek *et al*.[23]	+	+	–	+	+	+	–	+	–
Kubo *et al*.[24]	–	–	–	–	+	–	+	–	–
Abdalla and Garcia-Gody[25]	–	–	–	–	+	–	+	+	+
Loguercio *et al*.[26]	+	+	–	+	–	–	+	–	+
Burrow and Tyas[27]	–	+	–	–	–	–	–	+	–
Kurokawa *et al*.[28]	–	–	–	–	+	–	+	+	–
Loguercio *et al*.[29]	+	+	+	+	–	–	+	+	+
Sugizaki *et al*.[30]	–	–	–	–	+	–	+	+	–
Peumans *et al*.[31]	+	+	–	+	+	+	–	+	–
van Dijken *et al*.[32]	–	–	–	+	–	–	+	+	+
Pollington and van Noort[33]	–	–	–	–	–	–	+	+	–
Türkün and Celik[34]	–	+	–	–	–	–	+	–	–
van Dijken *et al*.[35]	–	–	–	+	–	–	+	+	–
Van Landuyt *et al*.[36]	+	+	+	+	+	+	–	+	–
Ritter *et al*.[37]	+	+	–	–	+	–	+	+	–
Kubo *et al*.[38]	–	–	–	+	+	+	+	+	–
Reis *et al*.[39]	–	+	+	+	–	–	+	+	+
Reis *et al*.[40]	+	+	+	+	–	–	+	–	+
Ritter *et al*.[41]	+	+	–	+	+	–	+	+	–
Wilder *et al*.[42]	+	+	–	+	+	–	+	+	–
Loguercio *et al*.[43]	+	+	+	+	–	–	+	–	+
Kubo *et al*.[44]	–	–	–	+	+	+	+	+	–
Yazici *et al*.[45]	–	–	–	–	–	+	+	+	–

**Figure 1:**
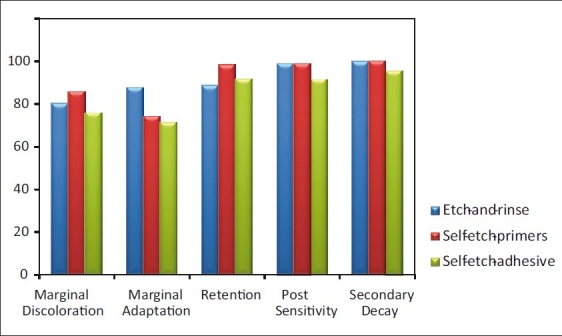
Mean Alfa score percentage of three categories of bonding agents

The Alfa score percentages reported in various studies is shown in Table 3. The mean Alfa score percentages for the different criteria of bonding agents are given in Table 4. There was no significant difference for retention, marginal discoloration, postoperative sensitivity, and secondary caries among the three categories [Figure 1]. However, there was a significant difference for a marginal adaptation between ER category and SEA category (*P*<0.05).

**Table 3:** Alfa score percentage cited in modified USPHS criteria for various studies

Adhesive category	Sample size (N/B^)	Recall period	Marginal discoloration	Marginal adaptation	Post-operative sensitivity	Retention	Secondary caries	Cumulative retention	Studies
Etch-and-Rinse									
Admira Bond	60/65	2 years	90.7	90.7	100	–	–	–	Abdalla and Garcia-Gody[25]
Single Bond	32/32	5 years	84	94	–	100	100		Kubo *et al*.[24]
+ QTH	48/77	3 years	68.7	68.7	–	–	100	84	Yazici *et al*.[45]
+ LED	48/77	3 years	77	77	–	–	100	84	
One step plus X4 coats	27/29	6 months	84	100	100	96.3	100		Loguercio *et al*.[26]
	27/29	6 months	60	86.7	100	77.8	100		
	23/31	2 years	73.9*	87*	100	100	100		Kubo *et al*.[38]
Clearfil LB	27/27	5 years	85	93	–	100	100		Kubo *et al*.[24]
Adper single bond	38/39	3 years	83.4*	83.4	100	96.7	100		Loguercio *et al*.[29]
	39/42	3 years	82	89.7	–	92.3			Reis and Loguercio[39]
Allbond 2	53.7%#	13 years						4.1$	van Dijken *et al*.[32]@
Clearfil LB	26.3%#	13 years						2$	
Denthesive	94.7%#	13 years						7.3$	
Gluma 2000	83.8%#	13 years						6.5$	
Gluma Solid bond + DS 1–2	25/26		100	92	–	100	100		Ritter *et al*.[37]
Opti-bond	40.6%#	13 years						3.1$	van Dijken *et al*.[35] @
Permagen	86.8%#							13.0$	
Scotchbond multipurpose	62.4%#							4.8$	
Syntac classic	36.4%#							2.8$	
Opti-bond FL	132/133	1 year	93.6	61.7	90.3	99.3	100		Van Landuyt *et al*.[36]
One step	39/42	3 years	74.3	87.2	–	51.4			Reis and Loguercio[29]
Opti-bond solo	43/48	3 years	88	98	98	98	100	93.3	Ritter *et al*.[41]
Prime and bond 2.1	46/51		88	100	100	91	100	89.4	
Opti-bond solo	29/48	8 years	45	60	100	69	100	65.6	
Prime and bond 2.1	27/51		69	100	100	59	100	60.6	
Opitbond dual cure	95/100	1 year	97	98	97	98	98		Wilder *et al*.[42]
	46/100	12 years	73	94	100	89	100		
Self-Etch primers (SEP)									
Clearfil SE Bond	50/50	2 years	93	59	98	100	100		Van Meerbeek *et al*.[23] @
+ Acid etch	50/50	2 years	95	80	95	100	100		
	60/65	2 years	92.3	92.3	–	100	–		Abdalla and Garcia-Gody,[25]
	50/50	5 years	68	17	100	98	100		Peumans *et al*.[31] @
+ Acid etch	50/50	5 years	83	52	100	100	100		
Tyrian + one step plus	27/29	6 months	80	92	100	96.3	100		Loguercio *et al*.[26]
4 × Tyrian + one step plus	27/29	6 months	75	87.5	100	96.3	100		
PUB 3	59.7%#	13 years						4.5$	van Dijken *et al*.[35]@
Clearfil protect bond + Filtek supreme	50/50	2 years	92	92	–	100	–		Türkün and Celik[34]
Clearfil protect bond + Dyract eXtra	50/50	2 years	92	96	–	94	–		
ART bond	41.3%#	13 years						3.2$	van Dijken *et al*.[32] @
Denthesive 2	74.3%#	13 years						5.7$	van Dijken[32] @
Self-Etch adhesives (SEA)									
Hybrid bond	57/65	2 years	64.6*	73.8	–	100	–		Abdalla and Garcia-Gody[25]
One-up Bond F	25/51	5 years	–	–	–	–	–	92	Burrow and Tyas[27]
Adper prompt L-Pop	21/21	1 year	100	52	100	100	100		Kurokawa *et al*.[28] 2007
	34/39	3 years	53.4	66.7	100	83.3	100		Loguercio *et al*.[29]
Prompt L-Pop + Pertac II	30/30	3 years	92.3	83.3	–	86.6	100		Pollington and van Noort[33]
+ Hytac	30/30	3 years	92.3	80	–	86.7	100		
AQ bond plus	21/21	1 year	100	33	100	100	100		Kurokawa *et al*.[28]
FB shake one	24/24	1 year	100	33	100	100	100		Kurokawa *et al*.[28]
G Bond	14/14	1 year	100	57	100	100	100		Kurokawa *et al*.[28]
	132/133	1 year	88.6	43.2	89.5	98.5	100		Van Landuyt *et al*.[36]
	54/55	2 years	79	100	–	98	100		Kubo *et al*.[38]
One up bond F+	18/18	1 year	100	44	100	100	100		Kurokawa *et al*.[28]
Xeno III (SEA)	30/30	18 months	100	100	100	100	100		Sugizaki *et al*.[30]
iBond + DS 1–2	26/28	3 years	69	81	–	100	100		Ritter *et al*.[37]
iBond + DS 3–4	20/25	3 years	35	70	–	100	100		
iBond + DS 3–4+ Etching	23/26	3 years	75	85	–	87	100		
PSA	56.6%#	13 years						4.4$	van Dijken *et al*.[35] @
Clearfil S3 bond	52/53	2 years	79	100	–	98	100		Kubo *et al*.[38] 2009
+ AP-X	46/49	3 years	76	96	–	100	100		Kubo *et al*.[44] 2010
+ flow FX	47/49	3 years	74	98	–	94	100		
+ Hydrophobic layer	30/30	18 months	53.3	70	73.3	76.6	76.6		Reis *et al*.[40]
	30/30	18 months	76.6*	83.3	86.6	93.3	93.3		
iBond Gluma inside	30/30	18 months	3.3	33.3	36.6	40	40		
iBond Gluma inside+ hydrophobic layer	30/30	18 months	56.6*	80*	80*	83.3*	83.3*		
All bond SE + all bond SE liner	28/33	2 years	66.6	72.7	100	84.4	100		Loguercio *et al*.[43]
	30/33	2 years	78.7	75.7	100	90.9	100		

@ Data adapted to fit modified USPHS criteria,

B^^^Number of subject at baseline,

* Statistically significant difference between groups,

# Lost to follow up,

$ Annual failure rates, DS, dentin sclerosis;

ER, etch-and-rinse; SEP, self-etch-primer; SEA, self-etch-adhesive; QTH, quartz tungsten halogen unit; LED, light-emitting diode unit; AP-X, hybrid composite; Flow FX, flowable composite.

**Table 4:** Mean Alfa score percentage for three categories of bonding agents

Adhesive category	Criteria	*N* (number of studies)	Min%	Max%	Mean %	± SD%
Etch-and-rinse (ER)	Marginal discoloration	19	45.00	100.00	80.15	13.25
	Marginal adaptation	19	60.00	100.00	87.43^a^	12.34
	Retention	16	51.40	100.00	88.61	15.74
	Post–op sensitivity	12	90.30	100.00	98.77	2.84
	Secondary caries	16	98.00	100.00	99.87	.50

Self-etch-primer (SEP)	Marginal discoloration	9	68.00	95.00	85.59	9.55
	Marginal adaptation	9	17.00	96.00	74.20	26.56
	Retention	9	94.00	100.00	98.29	2.26
	Post-op sensitivity	6	95.00	100.00	98.83	2.04
	Secondary caries	6	100.0	100.00	100	0

Self-etch-adhesive (SEA)	Marginal Discoloration	24	3.30	100.00	75.55	23.60
	Marginal adaptation	24	33.00	100.00	71.29^b^	21.92
	Retention	24	40.00	100.00	91.69	13.17
	Post-op sensitivity	13	36.60	100.00	91.23	18.31
	Secondary caries	23	40.00	100.00	95.36	13.44

The adhesives tested in these studies are listed with the ADA full acceptance in Table 5. SEA were tested more frequently. Among the 17 ER bonding system studied in the reported trials, four bonding systems did not satisfy the ADA specification full acceptable criteria. All the four bonding systems (Scotchbond Multipurpose, One Step, Gluma 2000, and Denthesive) belonged to the two-step ER type. Among the 18 studies involving the two-step SEA (SEP, 6 agents) and one-step SEA (SEA, 12 agents), two (Denthesive 2 and Tyrian SPE), and three (Hybrid Bond, iBond Gluma inside, and PSA Dyract) bonding systems, respectively, did not satisfy ADA specification full acceptable criteria.

**Table 5:** List of bonding agents tested in clinical studies with chemical composition and ADA acceptance

Material	Composition	ADA full acceptance
Etch and rinse		
Admira Bond (VOCO, Cuhaven, Germany)	Etchant: 36% phosphoric acid	Yes[25]
	Adhesive: Acetone, bonding ormocer, dimethacrylates, initiators, stabilizers.	
Scotchbond Multi-Purpose (3M ESPE, St.Paul, Minn.USA)	Conditioner: 10% maleic acid	No[35]
	Primer: 40% HEMA, 13% polyalkeonic acid copolymer with methacrylate groups, water	
	Adhesive: HEMA, bis-GMA, hexafluorophosphate, photoinitiator	
Adper Single Bond (3M ESPE, St.Paul, Minn. USA)	Scotchbond (3M ESPE): 37% phosphoric acid	Yes[29,39]
	Adhesive: Bis-GMA, HEMA, dimethacrylates, polyalkenoic acid copolymer, initiators, water, ethanol	
Clearfil LB (Kurary, Osaka, Japan)	Conditioner: 10% Citric acid, 20% CaCl2, 6 % Colloidal silica thickener, water.	Yes[24]
	Primer: 3% 5-NMSA (N-methacryloxy 5-aminosalisic acid)	
	Bonding agent: 10-MDP, bis-GMA, HEMA, Photoinitiator.	
All Bond 2 ((Bisco, Schaumburg, IL, USA)	Etchant: 32% phosphoric acid	Yes[32]
	Primer A: 2% NTG-GMA (N-p-tolyl-glycine-glycidyl methacrylate), photoinitiator.	
	Primer B: 16% BPDM (biphenoldimethacrylate), photoinitiator, ethanol, acetone.	
	Adhesive: bis-GMA, UDMA, 2-HEMA.	
Gluma 2000	Etchant: Oxalic acid 6.1%, aluminium nitrate 2.6%, glycine 2.7%, water (pH 1.3)	No[32]
	Primer/ Adhesive: N-methacryloxyethyl-N-methylformamide, bis-GMA, acetic acid, ethanol.	
One Step (Bisco, Schaumburg, IL, USA)	Uni-etch: 32% Phosphoric acid	No[29]
	Adhesive: Bis-GMA, BPDM, HEMA, initiator and Acetone.	
One Step Plus (Bisco, Schaumburg, IL, USA)	Uni-etch: 32% Phosphoric acid	Yes for retention, No for marginal discoloration[26,38]
	Adhesive: HEMA, BPDM, photoinitiator, Dental glass	
Optibond Dual Cure (Kerr, Orange, CA, USA)	Etchant: 37% phosphoric acid	Yes[42]
	Primer:2-Hydroxyethyl methacrylate (HEMA), Glycerol phosphate dimethacrylate, Mono (2-methacryloxyethyl) phthalate, Ethanol, Water	
	Adhesive: Catalyst resin liquid Bisphenol A glycidyldimethacrylate, HEMA, Chemical and light-cure catalyst	
	Accelerator paste: 48 percent filled by weight	
	Barium aluminum borosilicate glass, Fumed silica, Disodium hexafluorosilicate, Barium borosilicate glass, HEMA, Glycerol dimethacrylate	
Optibond FL (Kerr, Orange, CA, USA)	Etchant: 38% Phosphoric acid	Yes[36]
	Primer: HEMA, glycerol phosphate dimethacrylate (GDMA), mono-2-methacryloxyethyl phthalate (MMEP), water, ethanol, Camphoroquinone, butylhydroxy toluene	
	Adhesive (Optibond dual cure):	
	A activator: Bis-GMA, HEMA, GDPM, catalyst (benzoyl peroxide and camphoroquinone)	
	B paste: filler (fumed SiO_2_, Ba-Al-B-Si, Na_2_SiF_2_), disodium hezafluorosilicate, HEMA, tertiary amine	
Permagen (Ultradent Prod Inc, Utah, USA)	Etchant: 10% phosphoric acid	No[35]
	Primer: A:NTG-GMA (N-tolyglycine-glycidil mehtacrylate)	
	B: proprietary hydrophilic resin, acetone.	
	Adhesive: 2-HEMA, bis-GMA	
Syntac Classic (Ivoclar-Vivadent, Schann, Liechtenstein)	Etchant: 36% phosphoric acid	Yes[35]
	Primer: 25% TEGDMA, 4% maleic acid, water	
	Adhesive: 35% PEGDMA (polyethylene glycol dimethacrylate), 5% gluteraldehyde, 60% water	
	Resin (Heliobond): 60% bis-GMA, 40% TEGDMA	
Single Bond (3M ESPE, St.Paul, Minn.USA)	Etchant: 37.5% phosphoric acid	Yes[24]
	Adhesive: HEMA, bis-GMA, water, ethanol, dimethacrylates, photoinitiator system, methacrylate functional copolymer of polyacrylic acid and polyitaconic acid	
Denthesive (Heraeus Kulzer, Wehrheim, Germany)	Etchant: 5% EDTA (2 NaOH to pH 4.5)	No[32]
	Primer A: methacryloxyethylmaleate, ethanol.	
	B: 2-HEMA, phosphate, ethanol (pH 2.3)	
	Adhesive: Highly filled dimethacrylate	
Gluma Solid Bond (Heraeus Kulzer Hanau, Germany)	Etchant: 20% Phosphoric acid, Pyrogenic silica, Blue dye.	Yes[37]
	Primer: Maleic acid, HEMA, Mod. Polyacrylic acid, water, ethanol.	
	Adhesive: Bis-GMS, TEGDMA, HEMA, Carboxylic acid, Filler 25% (Ba-Al-B-F-Si glass and pyrogenic silica)	
Prime and Bond 2.1	Etchant: 34% Phosphoric acid	Yes[41]
	Adhesive: BisGMA, PENTA-P, photoinitiator, cetylamine hydrofluoride, acetone	
OptiBond Solo	Etchant: 37% Phosphoric acid	Yes[41]
	Adhesive: Alkyl dimethacrylate resin, Barium aluminosilicate glass, Sodium Hexafluorosilicate, fumed silica, ethyl alcoho	

Self-Etch Primer (SEP)		
ART Bond (Coltene, Alstatten, Switzerland)	Primer A: 1.6% Maleic acid, NaF, water.	Yes[32]
	Primer B: 36% HPMA (hydroxypropyl methacrylate), 6.2% PMA (polymethacrylic oligomaleic acid), 47% 2-HEMA, water.	
	Adhesive: 44% isopropylidenbis, 7% PMA, 49% DMA (dioxaoctamethylendimethacrylate), bis-GMA, TEGDMA.	
PUB 3 (Denstply, Konstanz, Germany)	Primer: 30% HEMA, 6% PENTA (dipentaerythreitol pentacrylate phosphonate ester), ethanol.	Yes[35]
	Adhesive: 4.5% PENTA, 25% TEGDMA, HEMA, 0.5% gluteraldehyde, 50% UDMA, photoinitiator	
Clearfil SE (Kuraray, Tokyo, Japan)	Primer: 10-MDP, HEMA, hydrophilic dimethacrylate, CQ,	Yes[23,25,31]
	N,N-diethanol p-toludine, water	
	Adhesive: 10-MDP, Bis-GMA, HEMA, hydrophilic dimethacrylate, CQ, N,N-diethanol p-toludine, silanized colloidal silica	
Clearfil Protect Bond (Kuraray, Osaka, Japan)	Primer: 5% MDPB, MDP, HEMA, hydrophobiic dimethacrylate, photoinitiators, water	Yes[34]
	Adhesive: MDP, Bis-GMA, HEMA, hydrophilic dimethacrylate, dicamphoroquinone, NaF, silanized colloidal silica	
Denthesive 2 (Heraeus Kulzer, Wehrheim, Germany)	Primer A: 2% Maleic acid, water	No[32]
	B: 82% HEMA, 3.6% maleic acid-mono-methacryloyl-oxy-propylester, 3.6% methacrylated polycarboxylic acid, TEGDMA, photoinitiator, stabilisator, water	
	Adhesive: (Adhesive Bond II) 43.5% bis-GMA, 7% maleic acid-mono-2-methacryloxyethyl, 48.5% TEGDMA, photoinitiator.	
Tyrian SPE (Bisco, Schaumburg, IL, USA)	Self-etching Part A: 20-30% Ethanol	No[26]
	Self-etching Part B: 2-acryl amido-2-methyl propane sulfonic acid (30-50%); bis2-(methacryloxyethyl) phosphate GMA (5-15%); Ethanol (40-70%)	
	Adhesive: bis-GMA and BPDM (15-40%); HEMA (15-40%); dental glass (1-10%) and acetone (40-70%)	
Self- Etch Adhesive (SEA)		

Clearfil S3 Bond (Kuraray, Osaka, Japan)	Methacryloyloxydodecyl dihydrogen phosphate (10 MDP), 2-HEMA, bisphenol A, diglycidyl methacrylate, water, ethanol, silanated colloidal silica, camphorquinone, photoinitiator.	Yes[38,44]
G Bond (GC Corp, Tokyo, Japan)	4-MET, phosphoric acid monomer, UDMA, Acetone, Water, Silinated colloidal silica and initiator	Yes[28,36,38]
AQ Bond plus (Sun Medicals)	4-META, UDMA, MMA, Water, Acetone, initiator, p-toluenesulphonate, reductant	Yes[28]
Hybrid Bond (Vivadent, Schann, Liechtenstein)	MMA, 4-META, tri (2-hydroxyethyl)-isocyanurat-triacrylate(THIT), HEMA, Acetone, Water.	No[25]
All Bond SE (Bisco, Inc)	Part I: Ethanol, benzene sulfinate dehydrate	Yes for SEA+Liner[43]
	Part II: Bis(glyceryl 1,3 dimethacrylate, biphenyl dimethacrylate.	
	All Bond SE Liner (hydrophobic): Bisphenol A diglycidylmethacrylate, HEMA, glass frit.	
iBond Gluma inside (Heraeus Kulzer, Hanau, Germany)	4-MET, UDMA, glutaraldehyde, acetone, water, stabilizer, photoinitiator.	No[40]
Fluorobond Shake One (Shofu, Tokyo, Japan)	PRG, Fluoroaluminosilicate glass, 4-AET, 4-AETA, bis-GMA, HEMA, Water, solvent, initiator.	Yes[28]
One up Bond F+ (Tokuyama Corp, Tokyo, Japan)	MAC-10, HEMA, MMA, multifunctional methacrylic monomer, fluoroaluminosilicate glass, water, photoinitiator, arly borate catalyst	Yes[28]
PSA Dyract (Dentsply, Konstanz, Germany)	PENTA, TEGDMA, elastomeric urethane-modified bis-GMA resin, fluoride, acetone, photoinitiator	No[35]
Xeno III (Dentsply, Sankin)	Catalyst liquid: Methacryloyloxyethyl acid, UDMA, fluoride-releasing phosphozene monomer and photosensitizer.	Yes[30]
	Universal liquid: HEMA, Ethanol, Water, Microfilled particles.	
Adper Prompt L-Pop (3M ESPE, St.Paul, Minn.USA)	Liquid 1: Methacrylated phosphoric esters, Bis- GMA, initiators based on camforoquinone, stabilizers	Yes for retention, No for marginal discoloration[28,29]
	Liquid 2: Water, HEMA, polyalkenoic acid, copolymer, stabilizers	
Prompt L-Pop (3M ESPE, St.Paul, Minn.USA)	Water, methacrylated-phosphoric acid-HEMA ester, BAPO initiator, fluoride complex parabens	No[33]

## DISCUSSION

In a systematic review, Heintze[46] found that the results of bond strength tests did not correlate with laboratory tests that evaluated the marginal seal of restorations such as microleakage or gap analysis. The quantitative marginal analysis of Class V fillings in the laboratory was unable to predict the performance of the same materials *In vivo*. The review suggested that microleakage tests or the quantitative marginal analysis should be abandoned and research should focus on laboratory tests that are validated with regard to their ability to satisfactorily predict the clinical performance of restorative materials. Peumans *et al*.[12] reported the results of 32 clinical trials, and the current review on 23 trials is a clear indication of interest in understanding the clinical behaviour of adhesive materials.

Certain level of uniformity while conducting clinical trials will allow comparisons of the results with other studies, thus enabling current best evidence.[47] In some papers under the current review, the materials and methods were poorly described (insufficient information provided regarding patient selection and in-/exclusive criteria, recall rates, reasons of patient-drop out, inter-evaluator agreement, etc.). In addition, a large variety in study design (not uncommonly without a proper control or “gold standard”, a “paired-tooth” design, adequate randomization, a sample size calculation, a sufficiently long follow-up), was noticed in these clinical trials, which makes it difficult to compare the overall clinical performance of adhesives.

To increase the power of a class-V clinical trial, the study methodology must also be standardized better in the future. In many studies, patient-related factors, such as age, oral hygiene, occlusal loading and dentin sclerosis are more determining than any material property.[48,49] This patient factor can be ruled out by applying a balanced study design. In such a set-up, pairs of equal teeth (for instance, first and second premolar at the same side, left and corresponding right incisor, canine and premolar, respectively) with similar lesions are chosen in each patient and each tooth is assigned to one of the experimental treatments in a randomized manner.[48] Also an adequate number of patients, rather than restorations, are paramount to extend the results from the statistical sample to the population; statistical power analysis can help to determine the number of patients required. In addition, recall periods must be standardized more, evaluation criteria must be assessed by calibrated independent examiners following a standard index system, and recall rates and reasons for patient drop-out must be reported as well.

Retention, marginal integrity, and marginal discoloration (clinical microleakage) are usually the key parameter used to judge upon clinical effectiveness of adhesives and modified USPHS criteria allows such assessments. The use of uniform reporting criteria would enable comparison of clinical effectiveness. Although significant information was reported by some studies,[27,32,35] their retention rates could not be used for statistical analysis in the current review due to a different protocol to assess clinical effectiveness. Certain studies[23,31,36] which did not employ USPHS criteria could still be adapted to fit the criteria since the assessment criteria were similar.

In a comprehensive systematic review of contemporary bonding agents,[12] it was concluded that the clinical effectiveness of two-step ER adhesives was greater than that of one-step SE adhesives. However, the current systematic review found the clinical performance of different categories of bonding agents was similar. Postoperative sensitivity and secondary caries were not seen to be a clinical problem with any category of bonding systems. This finding may probably due to the selection of NCCL. NCCL have higher degree of dentinal sclerosis thus offering protection from sensitivity.[50] In general, all three categories of bonding systems had retention rates of 88.61–98.29%.

The ER category the mean Alfa score for retention was seen to be 88.61% which is a little lower than the other two categories. This observation is probably attributed to the studies involving ER have a longer follow-up period resulting in lesser retention. The lowest retention (51%) observed with One Step (Bisco).[39] This acetone-based material when applied, results in thin layers following solvent evaporation, which is more susceptible to inhibition of polymerization[51] and the amount of acetone[52] directly affects the bonding. Among the 17 clinical studies involving ER, three (Scotchbond MP, One Step, and Denthesive) bonding agents did not qualify the ADA full acceptance. The poor performance of Scotchbond MP[35] was due to the use of 10% maleic acid instead of 37% phosphoric acid as a dentin conditioner. A 94.7% lost to follow-up was reported for the Denthesive group following 13 years of clinical evaluation.[32] Results of a study with such a low recall rate have to be interpreted with caution. The failure of One Step was attributed to composition[12] which was addressed and reintroduced as One Step Plus.[45]

The number of studies related to SEPs reported is less with low follow-up period. Among the six reported SEP agents in the clinical studies, Denthesive 2 and Tyrian SPE did not fulfil the ADA full acceptance criteria. The reason for clinical failure of Tyrian SPE used with One Step Plus could be due to increased amount of solvent (ethanol) to promote ionization of acidic monomers, leading to entrapment of water and solvent, thus affecting the degree of conversion of polymer.[53] Denthesive 2 employs 2% maleic acid in the primer which may not produce effective etching of enamel/dentin.[32]

The clinical performance of SEA was satisfactory in all criteria except for marginal adaptation showing inferior results compared to ER category, however comparable to SEP. The reason for this could be attributed to one study involving five SEA bonding agents.[28] This study observed a marginal breakdown as early as 3 months and less than 50% had Alfa scores at the end of one year. In general, failure on marginal adaptation may be caused due to thermal and mechanical stresses in the oral environment,[54] viscoelastic property of the restorative material,[55] water sorption and hydrolysis along the tooth-restorative interface[56] and unique stress patterns at the cervical margin of the tooth.[57] However, this study could not reason their findings.

The Alfa score percentages for marginal staining and marginal adaptation in general were low when compared to ER and SEP categories. The relationship between marginal staining and marginal adaptation has been discussed in studies.[24,31,38] Approximately, 70% of the marginal discoloration was seen at the mesial and/or distal margins of the restoration, where it is difficult to access during finishing and polishing.[43] Hence, the cause of staining could have been the accumulation of stains at the marginal step and crevice and not microleakage.[24,36,38]

Among the 12 SEA tested, 4 (Hybrid Bond, iBond Gluma inside, Prompt L-Pop, and PSA Dyract) did not meet the ADA specification. Both Hybrid Bond and iBond Gluma inside contain methacryloxyethyltrimellitic anhydride (4-META) as an active monomer component. SEA containing 4-META get converted to dicarboxylic 4-MET in the aqueous medium leading to a pH of 1 with a potential to etch dental hard tissue.[58] Due to the absence of the hydrophilic bonding agent in SEA systems, following polymerization, these materials behave like semipermeable membranes leading to hydrolysis of the bond. The marginal discoloration of iBond Gluma inside was attributed to rapid hydrolysis of 4-methacryloyloxyethyl trimellitic acid– (4-MET-), which are temperature-dependent, and discoloration had appeared as early as 1 month.[59,60] SEA that contain PENTA as an acidic monomer (PSA Dyract) are known to hydrolyze overtime resulting in microleakage and compromise long-term bonding effectiveness.[61] However, Mjor and Toffenetti[62] have reported that narrow gaps, crevices, ditches, and microleakage adjacent to composite restoration do not lead to secondary caries. Therefore, monitoring marginal staining is recommended to extend the longevity of restoration as well as the teeth.

In terms of adhesion durability, 3-step ER is considered as the “gold standard” among all bonding systems.[22] The problems related to SEA arise due to[22] (1) they are too hydrophilic and act, even after polymerization, as semipermeable membranes; (2) because of the high solvent concentration, it is impossible to obtain an adhesive resin layer of adequate thickness and void from residual solvent; (3) during solvent evaporation, the monomer/water ratio may change and subsequently result in phase separations and blistering; and (4) the acidic components of these adhesives may also adversely interact with the initiator system of the composite and so weaken the bonding complex.

On the contrary, clinical performance of SEA comparable to ER system was observed in the present review. This could be attributed to the following reasons, which could help overcome the limitations of SEA:

1. Enamel bevel was placed in six of the studies [Table 2]. Unground enamel surfaces with the prismless structure are contaminated with oral fluid and covered with pellicle, which might prevent bonding between the adhesive and resin,[63] especially the efficacy of mild SEA.[64] Hence, the placement of enamel bevel could have enabled better etching of enamel yielding better retention rates.
2. Chemical composition of certain SEP (Clearfil SE Bond and Clearfil Bond Protect) contains 10-MDP as a functional monomer dissolved in water and ethanol with a pH around 2. This hydrophilic monomer improves the wetting on the moist surface[61] and in addition the hydroxyl groups chelate with calcium forming chemical bonds.[65] This leads to stabilization on the interfacial bond. These systems (Clearfil SE Bond/Bond Protect/S3 Bond, G Bond, All Bond SE, Fluorobond Shake One, One up Bond F+ and Xeno III) also contain silica nanoparticles which result in a thicker adhesive layer acts a flexible interface that relieves interfacial stress between shrinking composite and rigid dentin.[66] In addition, the presence of polyalkenoic acid copolymer (Adper Prompt L-Pop) can form Capolyalkenoate complexes at the superficial region of the hybrid layer and within the superficial 3 *µ*m of dentinal tubules,[8] which might stabilize the bonded interface by providing water stability and a stress-relaxing effect.[67]
3. While employing the self-etch system, a pretreatment using 37% phosphoric acid has been found to improve retention rates.[23,24,31]
4. Application of two coats of adhesive has been found to increase bonding efficacy.[26,27] Consecutive coats can promote removal of water and solvent and allow more resin uptake into the collagen fibril network.[68]
5. The hydrophobic layer application has shown to improve retention rates of self-etch systems.[40] By applying a nonsolvent hydrophobic adhesive layer over a surface that was treated with a SEA system, the concentration of the hydrophobic monomers increases. This, in turn, reduces water sorption at the adhesive layer. In addition, the increased adhesive layer thickness leads to a thicker and more uniform adhesive layer with lower concentrations of retained water and solvent, which is known to reduce the detrimental effects of polymerization shrinkage of resin-based composite restorations.[69,70]

It must be noted that the clinical studies included for review provide short-term success rates. It would be interesting to study the long-term success rates of the same clinical studies which will enlighten on the true performance of self-etch bonding systems. In addition, the so-called self-etching systems were introduced to minimize the number of critical clinical steps involved in bonding. However, it is noted that methods to overcome the limitations of self-etching systems tend to increase the number of clinical steps.

## CONCLUSIONS

With the available results of short-term clinical studies, it can be concluded that:

1. Early loss of retention may not be the main cause of clinical failure.
2. The clinical effectiveness of SEP and SEA are comparable to ER bonding systems.
